# Effects of recombinant adeno-associated virus-mediated CD151 gene transfer on the expression of rat vascular endothelial growth factor in ischemic myocardium

**DOI:** 10.3892/etm.2014.2079

**Published:** 2014-11-19

**Authors:** HAIRONG FU, JIAHUA TAN, QI’NAN YIN

**Affiliations:** 1 Division of Basic Medical Sciences, Chongqing Three Gorges Medical College, Chongqing 404000, P.R. China; 2 Endocrinology and Reproduction Laboratory, Gynecology Hospital of Freiburg, Freiburg D-79106, Germany

**Keywords:** CD151, myocardial infarction, vascular endothelial growth factor

## Abstract

The aim of this study was to observe the effects of cluster of differentiation (CD) 151 on the expression of vascular endothelial growth factor (VEGF) in ischemic myocardium by the injection of a recombinant adeno-associated virus (rAAV) vector carrying the human CD151 gene. A rat acute myocardial infarction model was established, and rAAV-CD151 was injected into the ischemic myocardium. Four weeks later, the ischemic myocardium was removed in order to detect the expression of exogenous CD151 mRNA by reverse transcriptase polymerase chain reaction. In addition, the expression of CD151 and VEGF was detected by western blot analysis to evaluate the effect of CD151 overexpression on VEGF expression. Four weeks after injection of the vector, exogenous CD151 mRNA was expressed in the myocardial tissues of the CD151 group, whereas it was not detected in sham surgery, model control or rAAV-green fluorescent protein (GFP) gene-treated groups. The expression levels of CD151 protein were significantly higher in the CD151 group compared with those in the other three groups (P<0.05). The VEGF expression level in the CD151 group was higher compared with those in the control and GFP groups (P>0.05). These results indicate that rAAV-CD151 effectively transfects rat myocardial tissues, and may promote angiogenesis of the ischemic myocardium, improve left ventricular function and increase VEGF expression to improve ventricular function.

## Introduction

The transmembrane 4 superfamily (TM4SF), which is ubiquitously expressed in the cells or tissues of mammals, predominantly regulates cell adhesion and migration. As the most important member of this family, cluster of differentiation (CD) 151 forms a CD151-α3β1/α6β1 functional complex by binding integrin α3β1 or α6β1 at extracellular loop-specific sites, thus regulating the proliferation, migration and adhesion of endothelial cells, as well as the signaling transduction pathways (1).

It has previously been demonstrated that CD151 promotes angiogenesis of a rat model of hindlimb ischemia (2). The injection of a recombinant adeno-associated virus (rAAV) vector carrying the CD151 gene into rat ischemic myocardium results in the expression of CD151 mRNA and protein, significant densification of local capillaries, facilitation of angiogenesis and improvement of ventricular function; however, the molecular mechanisms have yet to be elucidated (3). Therefore, the aim of the present study was to investigate the effects of CD151 gene transfer on vascular endothelial growth factor (VEGF) expression and the associated molecular mechanism.

## Materials and methods

### Materials

pZeoSV-CD151 plasmid, pAAV-green fluorescent protein (GFP) vector and *E. coli* DH5a strain were obtained from Polepolar Research Company (Beijing, China). Umbilical vein endothelial cells (ECV304) were purchased from the China Center for Type Culture Collection (Wuhan, China). The pAAV-CD151 plasmid was constructed by our group. Briefly, a pair of primers were designed using the PzeoSV-CD151 plasmid as the template. The sequences were as follows: forward: 5′-GAGATCTATGGGTGAGTTCAACGAG-3′; and reverse: 5′-GGAATTCCTCAGTAGTGCTCCAGCTTGAG-3′. The CD151 gene fragment was amplified by PCR and inserted at the downstream of the CMV promoter packaging plasmid pAAV. The recombinant plasmid pAAV-CD151 was then subjected to digestion, identification and sequencing. Polyvinylidene difluoride (PVDF) membrane was obtained from Invitrogen Life Technologies (Carlsbad, CA, USA). The hypersensitive enhanced chemiluminescence (ECL) kit was purchased from Santa Cruz Biotechnology (Santa Cruz, CA, USA). Rabbit anti-VEGF antibody was obtained from Sigma (St. Louis, MO, USA). Mouse anti-human CD151 antibody and β-actin antibody were obtained from Santa Cruz Biotechnology. Horseradish peroxidase-conjugated goat anti-rabbit immunoglobulin G (IgG) antibody was purchased from Sigma. The apparatus used herein included a western blotting system (Trans-Blot^®^ Turbo^™^ Transfer system; Bio-Rad, Hercules, CA, USA), western blotting color developing reagent (Pierce Biotechnology, Inc., Rockford, IL, USA) and GeneTools density scanning analysis software version 3.02.00 (SynGene, Frederik, MA, USA). Recombinant AAV (rAAV)-CD151 and rAAV-GFP viruses were each packaged and copied with human embryonic kidney epithelial cells (293 cells; obtained from the Pathology Institute of Chongqing Medical University, Chongqing, China) by the three-plasmid co-transfection method. The viral titers were measured using reverse transcriptase PCR (RT-PCR) (4).

### Establishment of the myocardial infarction model, grouping and gene transfer

Healthy adult, male Sprague Dawley rats (clean grade, weighing 200–250 g) were provided by the Experimental Animal Center of Chongqing Mecical University (Chongqing, China). All of the experimental procedures were performed in accordance with the National Institutes of Health Guide for the Care and Use of Laboratory Animals (5) and were approved by the Biomedical Ethics Committee of Chongqing Three Gorges Medical College (Chongqing, China). The rats were randomly divided into sham surgery, control, GFP and CD151 groups (n=6). The rats were anesthetized with 60 mg/kg pentobarbital and catheterized with ventilator-assisted breathing. A thoracotomy was performed and the heart was exposed. With the exception of the sham surgery group, the rats were subjected to ligation of the left anterior descending coronary arteries, which led to the vertex cordis and left ventricular anterior walls turning cyanotic. The cyanotic edges were vertically injected with 4×10^11^ viral genomes (6) rAAV-CD151 (CD151 group) or rAAV-GFP exogenous genes (GFP group) or normal saline (control group). Five sites were injected, 7 mm apart. The same volume of normal saline was injected in the control and the sham surgery groups. The thoracic cavity was then closed and the rats were attended to until recovery of spontaneous breathing. Finally, the rats were fed in the animal house after regaining consciousness.

### Detection of CD151 mRNA expression by RT-PCR

The rats were sacrificed 4 weeks following the surgery, and the myocardial tissues at the infarction edges were frozen in liquid nitrogen. ECV304 cells transfected with rAAV-CD151 were used as the positive control, and distilled water was used as the negative control. Total RNA from the myocardial tissues and ECV304 cells was extracted and quantified using UV-Vis spectroscopy. Then 2 μg total RNA was subjected to reverse transcription, and 1 μl of the product underwent PCR, using GAPDH as the internal reference. PCR primers used were as follows: human CD151, upstream, 5′-GAGGTCTATGGGTGAGTTCAACGAG-3′ and downstream, 5′-AATTCCTAGGCGTAGTC-3′, 799 bp; β-actin, upstream, 5′-GGAGAAGGACCCAGATC-3′ and downstream, 5′-GATCTTCATGAGGTA GTCAG-3′, 300 bp. PCR was performed in a total volume of 25 μl using a 7500 real-time PCR system (Invitrogen Life Technologies), under the following conditions: 5 min of pre-denaturation at 94°C and then 1 min at 94°C, 1 min at 60°C and 40 sec at 72°C, for a total of 30 cycles, followed by 3 min of extension at 72°C. Finally, 10 μl PCR product was subjected to 1% agarose gel electrophoresis, and the resulting images were analyzed.

### Detection of CD151 and VEGF expression by western blot analysis

The rats were sacrificed 4 weeks following the surgery, and the myocardial tissues of the injected regions were removed and homogenized in lysis buffer in an ice bath. Subsequently, the homogenates were centrifuged at 4°C, 12,000 × g for 30 min and the supernatant was collected. The protein concentrations were determined using the Coomassie brilliant blue method. Protein from each group (40 μg) was separated by 12% SDS-PAGE and then electronically transferred at 4°C to a PVDF membrane. The membrane was then blocked with TBS-T solution (10 mmol/l Tris-HCl pH 7.5, 100 mmol/l NaCl and 0.1% Tween-20) containing 5% skimmed milk powder. The PVDF membrane was incubated with the following primary antibodies at 4°C overnight: rabbit anti-human VEGF, mouse anti-human CD151 antibody and β-actin antibodies. It was subsequently incubated at room temperature for 2 h with the following: horseradish peroxidase-conjugated goat anti-mouse and goat anti-rabbit IgG secondary antibodies. Relative expression of CD151 and VEGF proteins was detected using ECL (developing and exposure was performed in accordance with the manufacturer’s instructions) and grayscale scanning using the gel imaging analysis system. All data were normalized to β-actin.

### Statistical analysis

The categorical data were expressed as the mean ± standard deviation. Relative optical density (OD) values of protein bands from the western blot analysis were determined using a gel imaging analysis system. The experiments for each group were performed in triplicate, and significant differences were analyzed by a Student’s t-test. The corrected OD values were subjected to univariate analysis of variance. The categorical data of two groups were compared using a Student’s t-test. P<0.05 was considered to indicate a statistically significant difference.

## Results

### Expression of exogenous CD151 mRNA

In the present study the CD151 gene was connected to a downstream coding HA gene fragment (30 bp) that did not affect the expression or function of CD151 protein. In the PCR, the upstream primer hybridized with the start segment of CD151 gene, while the downstream primer hybridized with the end segment of the HA gene. Therefore, the PCR product originated from exogenous CD151 mRNA.

**Figure 1:**
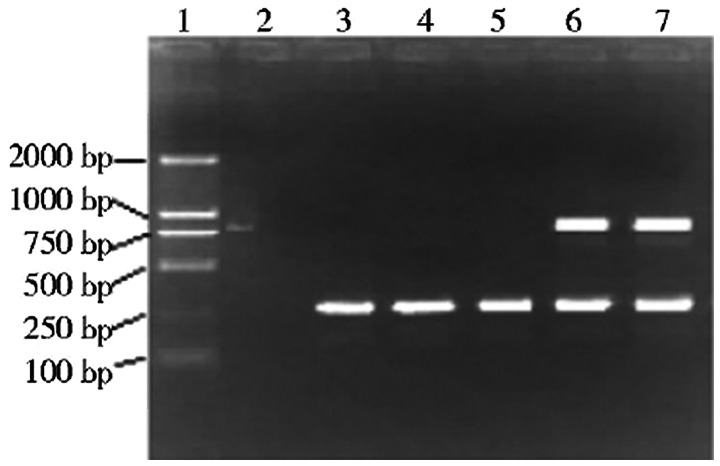
Expression of exogenous cluster of differentiation (CD) 151 mRNA in myocardial tissues. Lane 1: Marker; lane 2: negative control group (ddH_2_O); lane 3: sham surgery group; lane 4: control group; lane 5: green fluorescent protein group; lane 6: CD151 group; lane 7: positive control group (extracts of ECV304 cells).

The results from the RT-PCR (Fig. 1) demonstrated the presence of specific CD151 and β-actin bands in the CD151 and positive control (ECV304 cell extract) groups, indicating that the exogenous CD151 gene was expressed in the myocardial tissues of the CD151 group. Since only one band, corresponding to β-actin, was discerned in the sham surgery, control and GFP groups, exogenous CD151 gene was not expressed in these groups. Therefore, this indicates that the CD151 gene carried by the rAAV vector was stably and continuously expressed in the rats.

### Expression of CD151 protein

**Figure 2:**
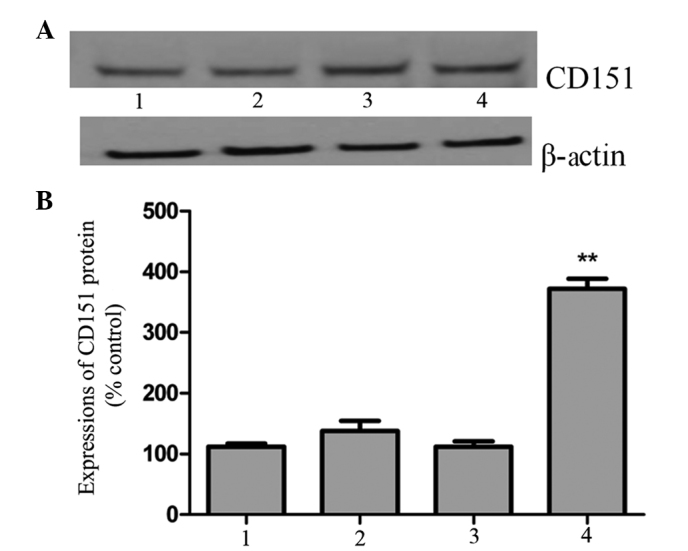
Expression of cluster of differentiation (CD) 151 protein. (A) the bands in the sham surgery, control and GFP groups were narrow and light, while in the CD151 group the band was broad and darker. (B) A significantly greater amount of CD151 protein was expressed in the CD151 group compared with that in the other three groups. Lane 1: Sham surgery group; lane 2: control group; lane 3: green fluorescent protein (GFP) group; lane 4: CD151 group. All data were normalized against β-actin and compared with those of the sham surgery group. ^**^P<0.05, versus the control and GFP groups.

There were specific bands observed in all groups four weeks following transfection with the CD151 gene. However, the bands in the sham surgery, control and GFP groups were narrow and light, while in the CD151 group the band was broad and darker (Fig. 2A). A significantly greater amount of CD151 protein was expressed in the CD151 group compared with that in the other three groups (P<0.05), while the outcomes of the sham surgery, control and GFP groups were similar (P>0.05; Fig. 2B). Hence, introduction of the human CD151 gene into rats significantly increased the expression of CD151 protein.

### Expression of VEGF

**Figure 3:**
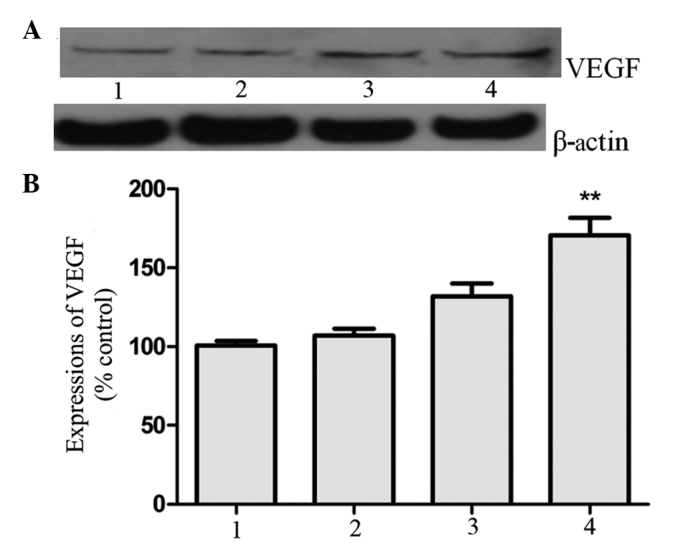
Expression of VEGF. (A and B) Elevated expression of CD151 was conducive to the expression of VEGF Lane 1: Sham surgery group; lane 2: control group; lane 3: green fluorescent protein group; lane 4: cluster of differentiation 151 group. The results from the sham surgery group were taken as 100%. ^**^P<0.05, compared with the sham surgery group. All data were normalized against β-actin and compared with those of the sham surgery group. VEGF, vascular endothelial growth factor.

VEGF expression was detected to elucidate whether VEGF could participate in angiogenesis in the CD151-induced rat myocardial infarction model. It was observed that the elevated expression of CD151 was conducive to the expression of VEGF (P<0.05; Fig. 3).

## Discussion

Angiogenic therapy has been investigated in basic and clinical studies on coronary artery disease. Revascularization has a crucial role in the recovery of cardiac functions following myocardial infarction. Angiogenesis in infarcted areas may mitigate the apoptosis of hypertrophic cardiomyocytes, maintain the activity of cardiomyocytes and inhibit collagen deposition, thereby protecting the heart and improving the prognosis. Angiogenesis is a complicated process leading to the development of new blood vessels, which involves the proliferation and migration of endothelial cells, regulation of the expression of proteolytic enzymes, reconstruction of degraded extracellular matrix and the formation of endothelial lumen (7).

As a highly specific, strong mitogenic factor for vascular endothelial cells, VEGF is able to predominantly induce physiological or pathological angiogenesis. By activating mitogen-activated protein kinase, stress-activated protein kinase, protein kinase C and the Akt pathway, VEGF induces organisms to produce proteases and specific integrins required for decomposition of the vascular basement membrane. As a result, cells are prone to proliferation, migration and survival, accompanied by angiogenesis. Furthermore, due to the activation of metalloproteinase, focal adhesion kinase and PI3K, endothelial cells are induced to migrate (8–10).

CD151, as the most important member of the TM4SF, is expressed in epithelial cells, endothelial cells, skeletal muscle cells, platelets, megakaryocytes and immature hematopoietic cells. In addition, CD151 participates in the adhesion, migration and proliferation of cells, as well as many physiological and pathological functions, including the interaction with integrin-mediated angiogenesis (11–14). It has previously been demonstrated that the rAAV-mediated expression of the CD151 gene in ischemic lower limbs and myocardial tissues is capable of promoting angiogenesis (14). However, the detailed mechanisms and whether VEGF is simultaneously highly expressed have yet to be established. Therefore, in the present study, western blot analysis was used to determine VEGF expression levels following myocardial infarction, in order to elucidate the likely effect on myocardial angiogenesis and the cardiac function of rats. It was observed that the transfection resulted in the upregulation of CD151 expression, and that the overexpression of CD151 facilitated VEGF expression, suggesting that VEGF expression is dependent on CD151.

In the present study, CD151 was demonstrated to upregulate the expression of VEGF, which may be responsible for enhanced angiogenesis of the ischemic myocardium. However, further investigation is required to determine if angiogenic molecules other than CD151 also have the same effect. The functions of CD151 and the underlying molecular mechanisms also require further study. The results in the present study provide information relevant to the treatment of cardiovascular diseases.
